# Vapor Selectivity of a Natural Photonic Crystal to Binary and Tertiary Mixtures Containing Chemical Warfare Agent Simulants

**DOI:** 10.3390/s20010157

**Published:** 2019-12-25

**Authors:** Joshua Kittle, Benjamin Fisher, Courtney Kunselman, Aimee Morey, Andrea Abel

**Affiliations:** Department of Chemistry, United States Air Force Academy, 2355 Fairchild Drive, Colorado Springs, CO 80840, USA; ben05fish@comcast.net (B.F.); cjkunselman18@tamu.edu (C.K.); aimee.morey@gmail.com (A.M.); andrea.abel.1@us.af.mil (A.A.)

**Keywords:** photonic crystal, mustard gas simulant, nerve agent simulant, principle component analysis

## Abstract

Vapor sensing via light reflected from photonic crystals has been increasingly studied as a means to rapidly identify analytes, though few studies have characterized vapor mixtures or chemical warfare agent simulants via this technique. In this work, light reflected from the natural photonic crystals found within the wing scales of the *Morpho didius* butterfly was analyzed after exposure to binary and tertiary mixtures containing dimethyl methylphosphonate, a nerve agent simulant, and dichloropentane, a mustard gas simulant. Distinguishable spectra were generated with concentrations tested as low as 30 ppm and 60 ppm for dimethyl methylphosphonate and dichloropentane, respectively. Individual vapors, as well as mixtures, yielded unique responses over a range of concentrations, though the response of binary and tertiary mixtures was not always found to be additive. Thus, while selective and sensitive to vapor mixtures containing chemical warfare agent simulants, this technique presents challenges to identifying these simulants at a sensitivity level appropriate for their toxicity.

## 1. Introduction

Positive identification of chemical warfare agents (CWAs) via passive, robust sensing remains an active area of research for the homeland defense, force protection, and treaty monitoring communities. Currently, mass spectroscopy and nuclear magnetic resonance spectroscopy are the primary means to identify CWAs at the parts per trillion level [1,2], though these techniques present challenges for portable, long-term, passive sensors suitable for field use. Piezoelectric, colorimetric, and electrochemical methods to detect CWAs and CWA simulants have been explored, but these techniques generally require improvements in selectivity, sensitivity, portability, and/or reusability before their wide-spread use as CWA sensors becomes practical [3]. However, photonic crystals (PhCs) could offer advantages over current detectors because of their potential improvements in cost, size, survivability, and simplicity as a continuous, passive sensor.

PhCs contain periodic structures comprised of at least two materials with different refractive indices and dielectric constants [4,5,6]. The simplest PhC is a one-dimensional Bragg reflector with many alternating layers of two different materials. Light incident on the Bragg reflector is both reflected and refracted at each material interface, leading to constructive and destructive interference of the light waves as they propagate through the PhC. Depending on the application, the materials composing the PhC and the periodic spacing between those materials can be used to generate photonic band gaps and structural color. Introduction of vapor into a PhC results in changes to the refractive index and periodic spacing of the structure (e.g., capillary condensation and/or swelling) [7,8,9]. These changes caused by the vapor result in detectable wavelength shifts of reflected light and thus enable sensing. This sensing technique is complex in that changes in the reflected light upon analyte introduction are caused by a number of factors, to include refractive index, vapor concentration, changes in structural periodicity, and vapor interactions with selective features within the PhC. These selective features include polarity gradients within natural PhCs and selective coatings or variable surface functional groups within synthetic PhCs [10,11,12,13,14]. High vapor sensitivity and visible light applications require spacing between structural features of the PhC to be in the range of hundreds of nanometers with large surface areas. While high surface area PhCs with selective features have been fabricated [12,15,16,17,18,19,20], these efforts are not trivial and widespread use of natural PhCs for sensing studies remains common.

The use of both natural and synthetic PhCs as vapor sensors has been widely explored over the last twenty years [5,6,7,8,9,10,11,12,13,14,15,16,17,18,19,20,21,22,23], though relatively few studies have examined their viability as CWA simulant detectors [24,25,26,27,28,29,30,31,32]. Typically, these studies have involved synthetic PhCs composed of porous silicon. Porous silicon PhCs without selective features (e.g., no functionalization) have detected the presence of CWA simulants via shifts in reflectance spectra with sensitivity as low as 4 ppm [27], though signatures were not unique and were a function of refractive index and concentration of the analyte vapor [27,28]. To introduce a degree of selectivity towards CWA simulants, porous silicon sensors have been fabricated with coatings containing functional groups reactive to CWAs and CWA simulants [25,26,29,30,32]. Generally, interaction of the CWA or CWA simulant with the coating caused a change in the thickness of the coating and thus the periodic spacing of the PhC, resulting in a detectable response that was restricted to analytes that react with the coating. However, most of these methods are susceptible to sensor poisoning and present challenges for use as a long-term passive vapor sensor for CWAs.

Establishing the viability of natural PhCs to discriminate between mixtures of CWA simulants is a relevant demonstration of the utility of PhC-based sensors as a low-cost, selective, sensitive, and robust means to passively and continuously sample and discriminate for these toxic vapors. A recent study by our group examined the viability of a natural PhC found in the wing scales of the *Morpho didius* butterfly to selectively distinguish CWA simulant vapors from other common vapors. The study showed that each of the five vapors analyzed yielded unique reflectance profiles, with sensitivity in the low hundreds of parts per million (ppm) [31]. Though an increasing number of works have examined both natural and synthetic PhCs as vapor sensors, very few works have studied the selectivity of PhCs to binary and tertiary vapor mixtures [11,12,23]. To our knowledge, no works have examined mixtures of vapors containing CWA simulants. 

In this work, the viability of a natural PhC to discriminate between binary and tertiary mixtures containing CWA simulant vapors was studied. Wing scales of the *Morpho didius* butterfly were exposed to vapors containing water, methanol (MeOH), ethanol (EtOH), and dichloromethane (DCM), as well as the CWA simulants 1,5-dichloropentane (DCP, mustard gas simulant) and dimethyl methylphosphonate (DMMP, nerve agent simulant). The wing scales were also exposed to binary and tertiary mixtures of water, MeOH, and EtOH and binary and tertiary mixtures of DCM, DCP, and DMMP. Reflectance spectra of each individual vapor and mixture were collected at several concentrations and the entire data set was processed using principle component analysis (PCA). Results indicated good vapor selectivity between all vapors and vapor mixtures studied with sensitivity down to the tens of ppm. However, binary and tertiary mixtures, while uniquely different from their individual vapors, did not always yield spectra bounded by their individual vapors, highlighting the complex response from the natural PhC.

## 2. Materials and Methods

The species *Morpho didius* is not subjected to any restriction. *Morpho didius*, a well-characterized species [33], was purchased from Butterfly Utopia and imaged via scanning electron microscopy (SEM). A forewing was flash frozen in liquid nitrogen and a 1 cm^2^ sample was cut from the center of the wing. This sample was then attached with carbon tape to a stub with the dorsal side up and sputtered-coated with 5 nm of gold. SEM images were then captured on the sputter-coated sample using a FEI Helios NanoLab 600 SEM operated at 5.0 kV.

For vapor sensing measurements, a fresh forewing was placed in a sealed glass flow chamber. A fiber optic reflectance probe (Ocean Optics, QR400-7-UV-vis) was placed normal to the wing. Light from a halogen source (Ocean Optics, HL-2000) was reflected off the center of the surface and collected via a spectrophotometer (Ocean Optics, HR2000+). A schematic of the experiment is provided in our previous work [31]. For all vapor measurements, a total gas flowrate of 400 mL·min^−1^ was used. Before each measurement, ultrahigh purity nitrogen (Airgas, UHP300) was used to flush the system for about 10 min to establish a stable reflectance profile. Once a stable reflectance profile was achieved, spectra for the PhC with nitrogen was captured and used as baseline for measuring differential reflectance (ΔR) upon introduction of an analyte vapor. To introduce an analyte, mass flow controllers were used to divert some of the nitrogen gas into a vapor saturation cell containing the liquid analyte. The pure nitrogen stream and analyte stream were then combined in a mixing chamber before entering the gas flow chamber. Partial pressure was used as the unit of concentration in this work, though it was not explicitly measured. Instead, by varying the flowrate of nitrogen carrier gas that entered the vapor saturation cell, the partial pressure of the analyte vapor was controlled and varied between 0.00 P_0_ and 0.50 P_0_, where P_0_ is the saturated vapor pressure of the analyte at 20 °C.

For binary and tertiary vapor mixtures, the vapor saturation cell contained 1:1 liquid by volume for binary mixtures and 1:1:1 liquid by volume for tertiary mixtures. The vapor concentration of each analyte in a mixture was calculated by assuming ideal mixtures and Raoult’s law. Spectral changes in response to analyte presence were typically visible in less than a minute, though spectra were collected after the signal stabilized (5 min). The range of concentrations for each analyte was run in series before testing a different analyte vapor. Nitrogen was run between analytes. Similar to previous work [31], a brief run with 0.15 P_0_ water vapor aided in quickly returning the response to the initial nitrogen baseline. The ΔR spectra were mean-centered in IgorPro, followed by PCA.

DMMP (97%, D169102) and DCP (99%, D69602) were purchased from Sigma-Aldrich, while EtOH (ACS grade, 111000190), MeOH (ACS grade, 339000000), and DCM (ACS grade, 313000ACSPL) were purchased from Pharmco-AAPER. Ultrapure water (Milli-Q gradient A-10, Milli-Q, 18.2 ΩM·cm, <5 ppb organic impurities) was used for all experiments.

## 3. Results

**Figure 1:**
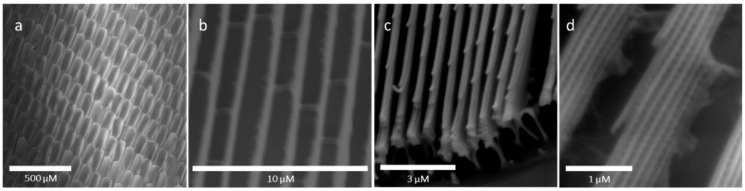
SEM images of the *Morpho didius* butterfly: (**a**) dorsal forewing scales; (**b**) ridges along the wing scales; (**c**) ridges with branch-like lamella visible; (**d**) side view of ridges with lamella and microribs visible.

As has been well-documented [33], the brilliant blue color of the *Morpho didius* arises from the complex nanostructure inherent within the wing scales. As shown in Figure 1a, the dorsal forewing of the *Morpho didius* is composed of many wing scales.

In turn, each scale contains about 70 ridges, each ridge less than 1 μm in width and running parallel to other ridges the length of the scale. Though difficult to observe in the images, these ridges are known to be thicker at the base (800 nm) than at the top (400 nm) and, as shown in Figure 1c, form a tree-like structure with six to eight lamellae forming the branch-like structures normal to each side of the ridge center [34]. These lamellae run the length of each ridge and are about 80 nm thick with about 150 nm vertical spacing between each lamellae layer. This gives a total height of each ridge of about 2 μm. Microribs, visible in Figure 1d, provide structural integrity by connecting lamellae within a ridge.

**Figure 2:**
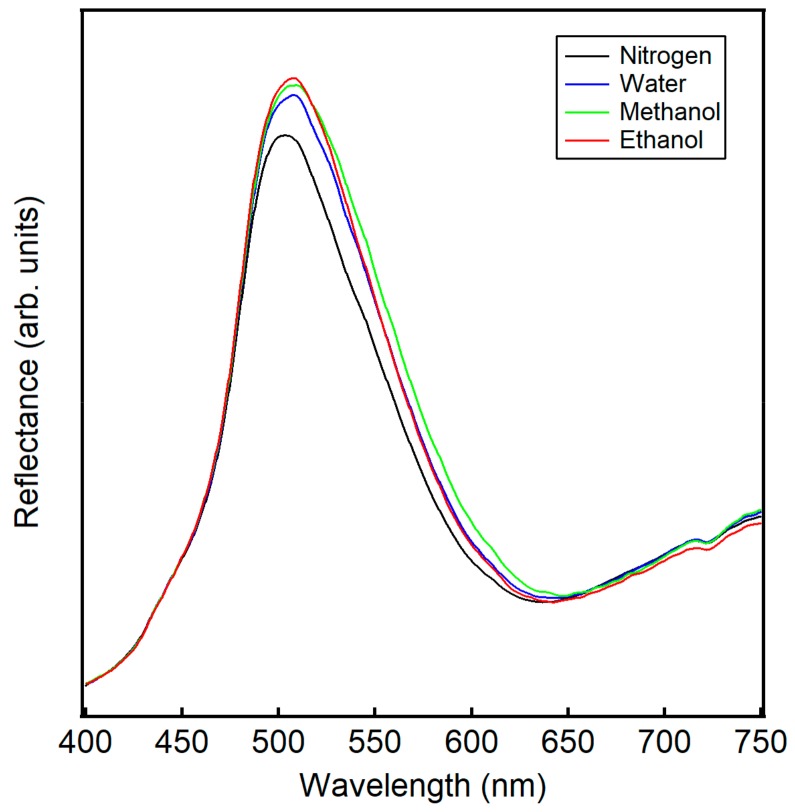
Raw reflectance data from incident light normal to wing scales of the *Morpho didius* in the presence of nitrogen (**−**), 0.50 P_0_ ethanol (**−**), 0.50 P_0_ methanol (**−**), and 0.50 P_0_ water (**−**). Note the shift in the reflectance peak upon the introduction of analyte vapor relative to nitrogen.

Light normal to the surface of the wing scales moves through the periodic structure of the ridges, lamella, and microribs, resulting in a photonic band gap (e.g., structural color) centered at about 500 nm, as shown for the measured reflectance in the presence of nitrogen in Figure 2. This reflectance peak is dependent on the angle of the light onto the PhC surface, as changing the angle changes the path length of the light [35]. The peak shifts for ethanol, methanol, and water in Figure 2 are distinct from one another due to differences in vapor concentration, refractive index, and polarity. Previous work Potyrailo et al. demonstrated that polarity significantly contributes to the selectivity of natural PhCs [11]. PhCs found in the wing scales of *Morpho* butterflies contain a polarity gradient, leading to analyte aggregation in particular regions and contributing to unique reflectance responses. While this phenomena are now well established, very few studies have examined the response of PhCs to binary and tertiary vapor mixtures.

**Figure 3:**
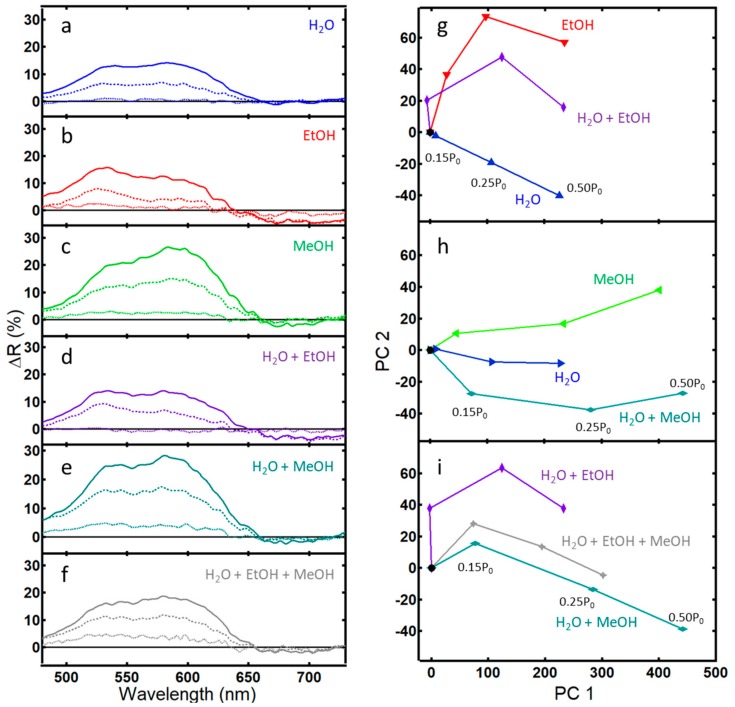
Differential reflectance data (ΔR) and corresponding PCA plots for light reflected from the PhC within wing scales of the *Morpho didius* butterfly: (**a**–**f**) ΔR after 5 min of exposure to the indicated vapor at concentrations 0.15 P_0_ (···), 0.25 P_0_ (--), and 0.50 P_0_ (—); (**g**) PCA scores plot of a binary vapor mixture of water and ethanol that captures 92.3% of the variance (PC 1 = 81.6%); (**h**) PCA scores plot of a binary vapor mixture of water and methanol that captures 96.8% of the variance (PC 1 = 94.8%); (**i**) PCA scores plot of a tertiary vapor mixture of water, ethanol, and methanol that captures 95.7% of the variance (PC 1 = 92.8%).

Differential spectra for single, binary, and tertiary vapors of water, ethanol, and methanol are shown in Figure 3a–f for concentrations of 0.15 P_0_, 0.25 P_0_, and 0.50 P_0_. The differential spectra, ΔR, is defined as:

(1) $$\Delta R=\;\left(\frac{R_{analyte}}{R_{blank}}-1\right)\;\cdot 100\%$$

where *R_analyte_* is the arbitrary reflectance of the analyte and *R_blank_* is the arbitrary reflectance of the blank (e.g., nitrogen).

Again, concentrations of the vapors are based on the partial pressure of the liquid. By assuming ideal mixtures, the contribution of an individual analyte can be calculated from Raoult’s law. Generally, vapor concentrations of water, ethanol, and methanol are of the same order of magnitude and in the thousands of ppm, with methanol having the highest vapor concentration and also the highest ΔR peak. Note, though, that traces of the individual vapors are unique, having peaks and shoulders in different areas. For example, while the overall traces for ethanol, methanol, and water vapor in Figure 2 appear visually similar, the difference in peak maximum, peak width, and the x-y shift relative to nitrogen give rise to the subtle differences in the ΔR traces in Figure 3a–c. Consistent with Figure 2 and more easily seen in Figure 3a–c, ethanol has the highest peak near 500 nm relative to water and methanol. Similarly, the broader peak and x-y shift for methanol in Figure 2 relative to the other spectra give rise to a broad peak in the differential spectra in Figure 3c centered near 600 nm. In Figure 2, ethanol shows a higher peak maxima than methanol and water, in line with predictions in our earlier work and consistent with refractive index trends [31]. However, the overall spectral difference ΔR for ethanol is lower than that of methanol or water as judged by the area of the curves in Figure 3a–c. This smaller response of ethanol is documented in the literature and is attributed to a lower affinity of ethanol to the natural chitin substrate [7,8,11]. Vapor mixtures take on character from their individual components, perhaps easiest seen for the mixture of water and methanol in Figure 3e with the raising of the first peak near 525 nm, similar to the trace for water.

Principle component analysis (PCA) is a powerful method to highlight variation between spectra that appear visually similar [36]. PCA of the binary and tertiary mixtures of water, ethanol, and methanol are shown in Figure 3g–i. All three data sets exhibit good selectivity and sensitivity [36]. The optical response of a binary mixture of water and ethanol fell between the pure analytes (Figure 3g). Similarly, data for a tertiary mixture of water, ethanol, and methanol fell between the responses of the binary mixtures (Figure 3i).

However, the optical response for a binary mixture of water and methanol did not fall between the values of the pure components. Inspection of Figure 3a–e shows that, while ΔR for a binary mixture of water and ethanol is additive from its individual components, this is not the case for the binary mixture of water and methanol. Instead, the overall ΔR for the water and methanol mixture remains closer to the value of methanol rather than lowering to a value closer to water as might be expected. A poor response of natural PhCs towards methanol and water mixtures has been previously noted in the literature [12], highlighting one of the challenges of sensing via this technique—a non-additive response.

**Figure 4:**
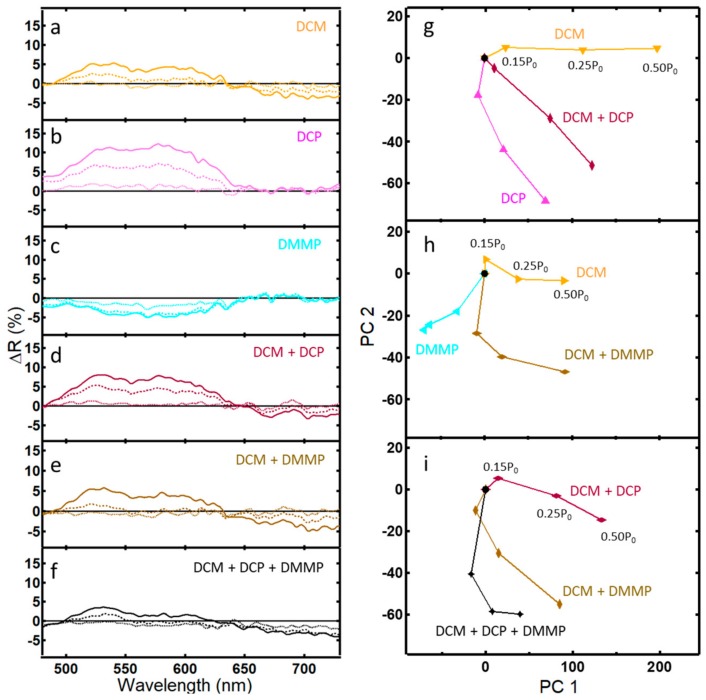
Differential reflectance data (ΔR) and corresponding PCA plots for light reflected from the PhC within wing scales of the *Morpho didius* butterfly: (**a**–**f**) ΔR after 5 min of exposure to the indicated vapor at concentrations 0.15 P_0_ (···), 0.25 P_0_ (--), and 0.50 P_0_ (—); (**g**) PCA scores plot of a binary vapor mixture of DCM and DCP that captures 84.9% of the variance (PC 1 = 72.9%); (**h**) PCA scores plot of a binary vapor mixture of DCM and DMMP that captures 80.9% of the variance (PC1 = 73.2%); (**i**) PCA scores plot of a tertiary vapor mixture of DCM, DCP, and DMMP that captures 78.9% of the variance (PC 1 = 58.8%).

Differential spectra for single, binary, and tertiary vapors of DCM, DCP, and DMMP are shown in Figure 4a–f for concentrations of 0.15 P_0_, 0.25 P_0_, and 0.50 P_0_. Since water is a poor solvent for DCP and DMMP, DCM was instead used to create equal-volume liquid binary and tertiary mixtures. Again, by assuming ideal mixtures, the contribution of an individual analyte can be calculated from Raoult’s law. Due to their different saturated vapor pressures, vapor concentrations of DCM are in the thousands of ppm, while the vapor concentrations of DCP and DMMP are in the tens of ppm. Thus, as might be expected, DCM has a higher ΔR peak than DCP and DMMP. Similar to the previous set of analytes, the traces of the individual vapors are unique, having peaks and shoulders in different areas, and the optical response for binary mixtures appears to be additive.

PCA of the binary and tertiary mixtures of DCM, DCP, and DMMP are shown in Figure 4g–i. The optical response of a binary mixture of DCM and DCP fell between the pure analytes (Figure 4g). Similarly, data for a binary mixture of DCM and DMMP fell between the responses of the binary mixtures (Figure 3h). However, the optical response for a tertiary mixture of DCM, DCP, and DMMP did not fall between the values of the binary mixtures. Similar to the case of water and methanol above, a non-additive response instead occurs, presenting challenges to analyte identification that will be more fully discussed later.

**Figure 5:**
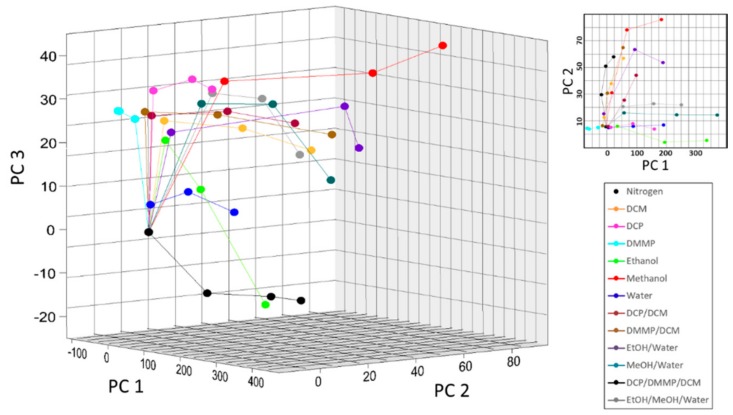
PCA score plots for individual and mixed vapors showing good selectivity, where the first three principle components (PCs) captured 93.0% of the total spectral variance (PC 1 = 87.5%, PC 2 = 4.2%, and PC 3 = 1.3%).

A PCA scores plot of all analytes tested in this work is shown Figure 5, where each data point in a trace corresponds to analyte concentrations of 0.15 P_0_, 0.25 P_0_, and 0.50 P_0_, respectively. While the optical response of the PhC to binary and tertiary mixtures were not always additive in this work, the system showed good selectivity and sensitivity to all analytes. Good selectivity was indicated by the distinct traces for each analyte evident in the three-dimensional scores plot [36]. This selectivity was evident despite similar refractive indices and concentrations, especially for mixtures containing CWA simulants because their vapor contributions were less than 0.2% by mass. Similar to previous work, no trends based on analyte polarity or refractive index were observed [31]. The sensitivity of the PhC was demonstrated by the spacing of individual data points within an analyte trace [36]. This sensitivity was evident over several orders of magnitude, from tens of ppm for CWA simulants up to tens of thousands of ppm for methanol. The CWA simulant DMMP was tested at concentrations as low as 120 ppm individually and 30 ppm in mixtures. The CWA simulant DCP was tested at concentrations as low as 240 ppm individually and 50 ppm in mixtures.

## 4. Discussion

This work demonstrated several advantages to using PhCs for CWA simulant detection. Most importantly, this technique showed selectivity to single, binary, and tertiary vapor mixtures with similar polarities and refractive indexes over a wide range of individual concentrations. Few studies have examined reflectance from PhCs upon introduction of binary or tertiary mixtures, though those studies also showed selective responses to analytes [12,23]. A key result of this work, however, was that introduction of even small vapor mass percentages (less than 0.2%) of CWA simulants into a vapor mixture resulted in significant, detectable shifts in reflectance spectra. Further, as shown in Figure 5, all vapors and mixtures analyzed showed good selectivity in that each PCA trace is distinctly separate in three-dimensional space [36]. In agreement with other studies, the response to introduction of analyte (e.g., deviation from the baseline) was fast and generally less than one minute. The natural PhC substrate did not irreversibly react with any of the tested analytes, as evidenced by the rapid return of the reflectance signal to its baseline upon reintroduction of pure nitrogen gas. This result was found in a number of other studies using a variety of natural PhCs and analytes, confirming the robust and reusable nature of PhCs for vapor sensing, to include CWA simulants [10,11,22,23,27,28,31]. Collectively, this work showed that monitoring light reflectance from a natural PhC is a viable method to quickly and reversibly detect changes to a vapor environment that contains trace amounts of a CWA simulant and a mixture of other vapors.

However, while this method was shown to be a rapid, reversible, passive means to generate unique responses to binary and tertiary vapor mixtures containing CWA simulants, several challenges remain for practical application of this method to sensor development. First, the sensitivity of this method towards DMMP, a nerve agent simulant, is inadequate, while the sensitivity towards DCP, a mustard gas simulant, is marginal. This work showed a unique reflectance with introduction of about 30 ppm DMMP and 50 ppm DCP. To date, the lowest sensitivity reported for DMMP vapor using a PhC is 4 ppm [27]. Due to the inherent toxicity of CWAs, sensitivities in the ppb are required. Thus, to be a viable sensing method for CWAs, the sensitivity must be improved by several orders of magnitude.

A second challenge to using PhCs as vapor sensors is that, while each analyte tested yielded a unique response, that response was not always additive for binary and tertiary mixtures. This is clearly seen in the PCA plots found in Figure 3h and Figure 4i. While studies have generally reported additive results for spectra from vapor mixtures [12,23], one study noted poor selectivity of a natural PhC to mixtures of water, methanol, and ethanol, a result confirmed here in that this work also did not yield a strictly additive result of the individual spectra [12]. This underscores the complexity of the response of natural PhCs to analyte vapors.

The non-additive response for vapor mixtures reported here and elsewhere has not been satisfactorily answered in the literature and presents opportunities for further study. Again, the spectral response is a function of the refractive index of the analyte, as well as its concentration and polarity [11]. A vapor mixture contains multiple analytes, each with unique refractive indices, concentrations, and polarities competing for binding sites at the variably polarized surface of the natural PhC. Work by Potyrailo et al. suggests that the cause for the non-additive response is due to differences in the material surface of the photonic structure because a natural photonic crystal yielded poor selectivity to mixtures of alcohol vapors, while a surface-functionalized natural photonic crystal resulted in improved responses to these mixtures [12]. However, numerical or experimental studies of photonic crystals with the same material and variable structure compared to studies of photonic crystals with the same structure and variable material could be useful for clarifying the underlying causes of non-additive responses for some vapor mixtures.

Regardless, this non-additive response presents challenges in identifying an unknown analyte. For example, the reflectance response of a class of analytes, such as phosphonates, may vary widely within that class and introduction of additional vapors could significantly alter and mask the identity of the original analyte of interest. Thus, a large library of analyte responses at various concentrations and vapor mixtures to determine the identity of an unknown appears impractical.

There are a number of methods to address these challenges, though tackling both sensitivity and selectivity will almost certainly require a synthetic PhC or pairing with another sensing method, such as surface acoustic wave-based sensors [12]. Improving sensitivity will require careful design of a synthetic PhC optimized for refractive index difference with the analyte of interest, quantity and spacing of structural features within the PhC, and operation in stimulated emission regimes, along with concentration of the analyte with time [10,11,12,31]. Due to the complex response to analytes and mixtures, a PhC will likely need to be tailored for reactive, selective response to a single analyte of interest. Alternatively, an array of natural and/or variably functionalized PhCs could be used to generate a small library of responses to environmentally relevant mixtures and concentrations to yield a high confidence in identifying an analyte of interest (e.g., mustard gas).

## 5. Conclusions

This work demonstrated that light reflected from natural photonic crystals found in the wing scales of the *Morpho didius* yield unique spectra when exposed to binary and tertiary mixtures of chemically and physically similar vapors of interest. However, the response to these mixtures was not always additive and the sensitivity towards the chemical warfare agent simulants was in the tens of ppm. Thus, while natural photonic crystals offer a means to rapidly, reversibly, and passively detect an analyte vapor, challenges remain in using this technique to identify an unknown vapor at relevant concentrations, especially for toxic materials. However, an array of natural or synthetic photonic crystals, designed for increased sensitivity or coupled with another sensing technique, could address the challenges of identifying low concentrations of chemical warfare agent simulants in vapor mixtures.
